# Investigation of Freeway Incident Duration Using Classification and Regression Trees Based on Multisource Data

**DOI:** 10.3390/s24227225

**Published:** 2024-11-12

**Authors:** Xun Xie, Gen Li, Lan Wu, Shuxin Du

**Affiliations:** 1 College of Automobile and Traffic Engineering, Nanjing Forestry University, Nanjing 210037, China; xiexun@njfu.edu.cn (X.X.); ligen@njfu.edu.cn (G.L.); wulan@njfu.edu.cn (L.W.); 2 Huzhou Key Laboratory of Intelligent Sensing and Optimal Control for Industrial Systems, School of Engineering, Huzhou University, Huzhou 313000, China

**Keywords:** machine learning, CART, incident duration, multisource, sensor data

## Abstract

Targeted contingency measures have proven highly effective at reducing the duration and harm caused by incidents. This study utilized the Classification and Regression Trees (CART) data mining technique to predict and quantify the duration of incidents. To achieve this, multisensor data collected from the Hangzhou freeway in China spanning from 2019 to 2021 was utilized to construct a regression tree with eight levels and 14 leaf nodes. By extracting 14 rules from the tree and establishing contingency measures based on these rules, accurate incident assessment and effective implementation of post-incident emergency plans were achieved. In addition, to more accurately apply the research findings to actual incidents, the CART method was compared with XGBoost, Random Forest (RF), and AFT (accelerated failure time) models. The results indicated that the prediction accuracy of the CART model is better than the other three models. Furthermore, the CART method has strong interpretability. Interactions between explanatory variables, up to seven, are captured in the CART method, rather than merely analyzing the effect of individual variables on the incident duration, aligning more closely with actual incidents. This study has important practical implications for advancing the engineering application of machine learning methods and the analysis of sensor data.

## 1. Introduction

**Figure 1:**
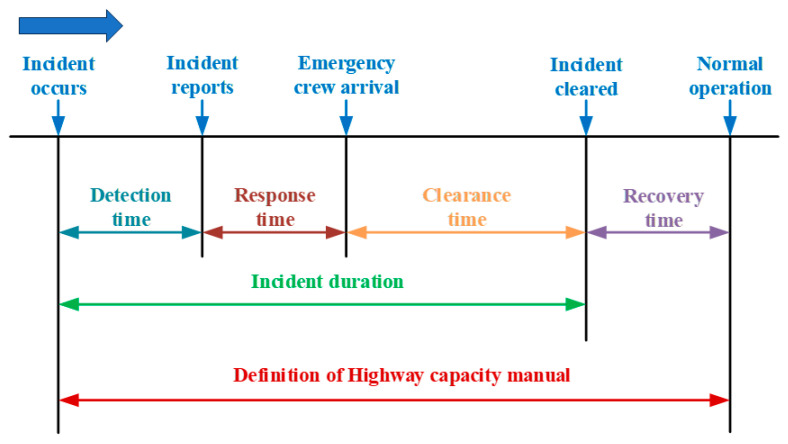
Concept of incident duration.

Extended incident durations have the potential to cause traffic congestion and secondary incidents. The duration of incidents is typically classified into four phases according to the *Highway Capacity Manual*: detection time, response time, clearance time, and recovery time. Previous studies on incident duration have commonly defined it as the time from incident occurrence to clearance, incorporating detection, response, and clearance times [1,2], as shown in Figure 1. The investigation of freeway incidents, particularly those that occur infrequently but have prolonged durations, is crucial for the enhancement of incident management strategies and the mitigation of incident-related harm. Moreover, the development of tailored contingency measures, based on specific incident information in different scenarios, can effectively facilitate the practical implementation of incident duration research in engineering applications and contribute to the reduction in incident duration.

In the previous literature on incident duration, much emphasis has been placed on predicting the duration of incidents [3,4,5]. The primary research methods employed include statistical models and machine learning models. Statistical models have been widely employed due to their rigorous mathematical formulas and strong interpretability [6,7,8]. Early regression models and their various enhanced methods have been used in the study of incident duration [9,10]. However, assuming a linear relationship between influencing factors and incident duration is inadequate. Hazard-based methods offer the ability to capture the duration effect and are commonly utilized in incident duration research [11,12,13]. Chung developed an accelerated failure time metric model (AFT) to predict the duration of incidents on a Korean freeway [14]. The model’s temporal transferability was validated using data from the second year. Hojati et al. constructed an AFT survival model for incident duration and concluded that the Weibull AFT model with random parameters is suitable for simulating the duration of incidents caused by collisions and hazards, while the Weibull model with gamma heterogeneity is the most appropriate for simulating the duration of stationary vehicle incidents [15]. In a study conducted by Islam et al. using incident duration data from Alabama freeways, a comparative analysis was performed between hazard-based random parameter duration models and latent class models. The findings indicated that the latent class model provided a better fit for the incident duration data [16].

In recent years, machine learning models have garnered attention for their enhanced accuracy [17,18,19,20,21]. Unlike statistical models, machine learning methods can handle highly complex relationships and achieve greater precision [22,23,24,25], albeit at the cost of weaker interpretability. Among these methods, the K-Nearest Neighbor (KNN) approach is widely utilized. Valenti et al. proposed an improved KNN model that incorporated a Euclidean-based distance function, leading to improved prediction accuracy through variable weighting [26]. Wen et al. introduced a novel distance measure and a method for determining weights in the KNN model, yielding favorable results in predicting traffic incident data in The Netherlands [27]. Similarly, support vector machine (SVM), another distance-based machine learning method, has also been extensively employed [28]. Neural network techniques are commonly utilized for incident duration prediction as well. Boyles et al. developed a probabilistic model based on a Bayesian classifier that performed admirably in predicting incident duration [29]. Wei and Lee proposed an adaptive model based on artificial neural networks and data fusion techniques, demonstrating robust predictive capability with most average absolute percentage errors below 40% [30]. In a comparison conducted by Yu et al. between artificial neural networks and support vector machines for incident duration prediction, it was found that the artificial neural network model outperformed the support vector machine model for longer duration incidents [31]. However, in overall incident duration prediction, the SVM model exhibited superior comprehensive performance to the artificial neural network model. Table 1 compares the current research progress and methods in the field of incident duration research.

Based on the previous discussions, machine learning methods that rely on distance measurement and neural networks are more suitable for predicting incident duration but have weaker explanatory effects on the influencing variables. In contrast, tree-based machine learning methods not only provide high prediction accuracy but also reflect the impact of explanatory variables. For instance, Zhan et al. [5] employed the M5P tree algorithm to enhance the prediction accuracy of incident clearance time and discovered that variables such as blocked lanes, time of day, and types and quantities of vehicles significantly influenced incident duration. He et al. proposed a quantile regression method based on mixed trees, demonstrating that incident characteristics (e.g., type, severity, blocked lanes, number of vehicles involved) were the most important predictors of incident duration [32]. Ma et al. [33] divided one year of incident duration data from Washington State into long and short duration groups using the gradient boosting decision trees (GBDT) algorithm and found that GBDT outperformed other algorithms. They also observed that incident response time had the largest impact on short duration incidents, while lane closure had the most significant impact on long duration incidents. Although these tree-based algorithms discuss the degree of influence of variables on incident duration while maintaining prediction accuracy, the results often function as pre-incident warnings. Consequently, discussing the importance of variables after incidents occur becomes meaningless. Therefore, it is necessary to classify incidents and establish comprehensive contingency measures. By implementing corresponding plans based on on-site conditions after an incident occurs, we can effectively reduce incident duration and achieve practical engineering applications. Furthermore, most of the aforementioned studies used traffic incident data from developed countries like the United States. However, due to differences in road conditions and management policies, the factors influencing incident duration may present different patterns in developing countries such as China. Hence, it is crucial to conduct localized research on incident duration.

In addition, road safety involves multifaceted factors that include road infrastructure (e.g., road surface conditions), road incident detection, traffic (e.g., congestion), and other environmental issues (e.g., inclement weather). Developing road safety strategies and action plans that address all these factors is challenging. Traditional approaches to road safety tend to guide relevant scientific knowledge and research, with the expectation that these tools will improve decision-making processes around the world [34,35,36,37], or focus more on transforming road users and designers [38,39,40,41,42]. As a result, a large body of research considers historical incident data and analyses incidents and other relevant data to predict traffic congestion [43], or to present critical analyses [44]. In recent years, sensor data have been widely used due to the advantages of high precision, high resolution, and strong self-adaptation. Its applications in the field of traffic safety include road condition monitoring [38,45,46,47], road user behavior monitoring and prediction [48,49,50], traffic congestion monitoring [51,52], and accident prediction or detection [53,54].

Using a large amount of sensor data from a typical freeway in China, this study proposed to model and analyze the incident duration via a machine learning method, namely the CART method, to address the aforementioned issues. CART, a tree-based method, is widely used for both classification and regression tasks. In this study, it was designed to address a primary problem: regression, where the aim was to predict continuous numeric values. The algorithm constructs a binary tree structure by partitioning the data into subsets based on the feature values, aiming to minimize the sum of squared residuals (the difference between the observed and predicted values) within each subset. In the tree, each branch represents a decision rule, and each leaf node represents a target value or class label [55,56]. Compared to other complex tree algorithms, CART provides stronger interpretability. The key advantage is that the corresponding rules can be extracted according to the fitting results of the CART method, and the targeted contingency measures can be proposed according to these rules. The incident duration can be reduced effectively by judging the situation of the actual incident and applying these measures. In addition, to further verify the validity of the CART method, the CART method was compared with XGBoost, RF, and AFT models. We also found that the actual incident was not affected by a single variable, but the superimposed effect of the interaction of several variables. In-depth analysis and discussion of this interaction in sensor data is of great significance to the practical engineering application of this research.

## 2. Data and Methods

### 2.1. Data

The dataset utilized in this study was obtained from the G2504 Hangzhou freeway, spanning a period of three years from 2019 to 2021. It includes 2927 records of traffic incidents, documented by sensors for vehicle detection, road condition detection, incident detection, environmental detection, and so on at the location of incidents. The study segment covers approximately 23 km, extending from the Hongken interchange to the Linpu interchange. This segment features a main line with a speed limit of 120 km/h, consisting of dual eight-lane carriageways and a roadbed width of 42 m.

**Figure 2:**
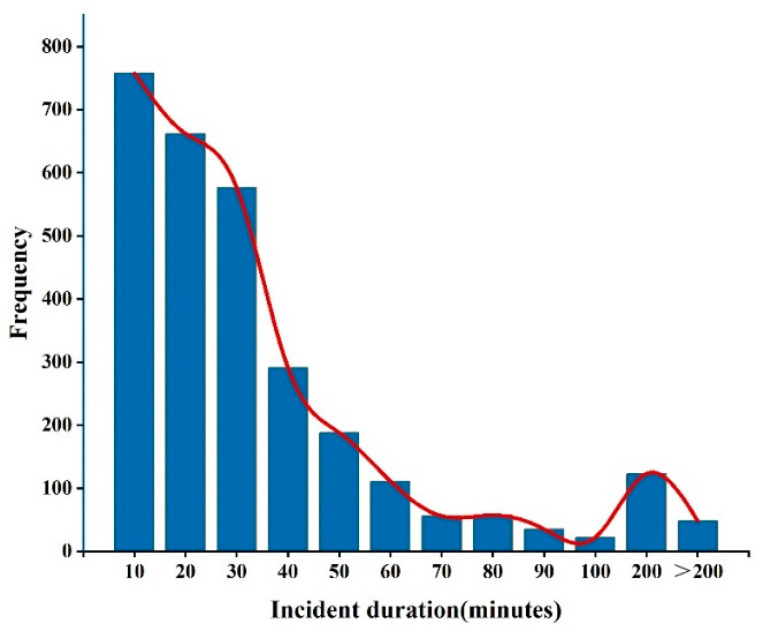
Distribution of freeway incident duration.

Table 2 presents the recorded information of each incident, encompassing incident characteristics, temporal characteristics, environmental characteristics, traffic characteristics, and operational characteristics. The duration of these incidents varies from 1 min to a maximum of 18 h and 48 min, with an average duration of 32.95 min and a standard deviation of 53.59 min. The distribution of incident duration is depicted in Figure 2. Of the 2927 recorded incidents, 1869 cases from 2019 to 2020 were used as the training set for the CART method, making up 64% of the dataset, with the rest used as the test set.

### 2.2. Methods

**Figure 3:**
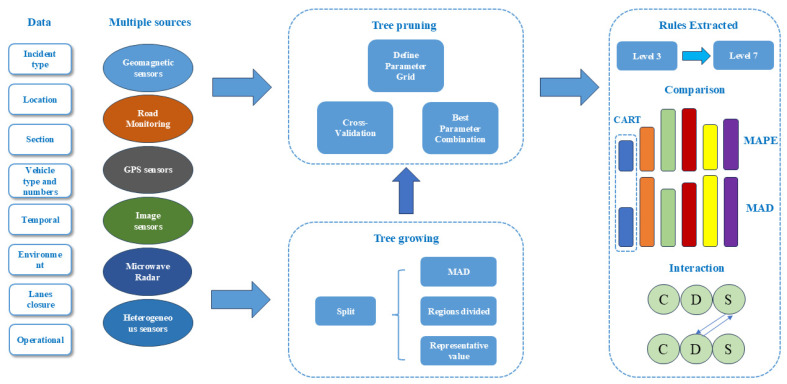
Overview of the overall methodology.

Regression trees in CART were selected for the prediction analysis of incident duration. The overall method of this paper is summarized as shown in the Figure 3. In the third panel on the right, different colors represent different models, which are CART, Weibull AFT, Log-normal AFT, Log-logistic AFT, XGBoost, and RF models.

#### 2.2.1. CART

**Figure 4:**
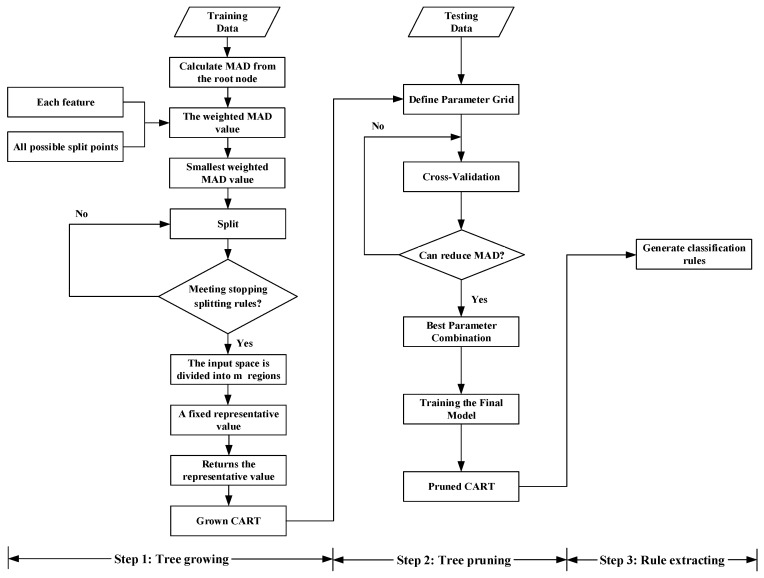
Building of regression tree in CART.

In the CART method, the root node encompasses all the records, while the leaves symbolize different groups. The tree construction process commences from the root node and traverses the path based on the attributes of each record, resulting in tree splits using criteria for attribute evaluation. The primary steps in model development encompass tree growing, pruning, and rule extracting, as demonstrated meticulously in Figure 4.

Given a training dataset $D=\left\{\left(X_{1},y_{1}),(X_{2},y_{2}),\ldots ,(X_{n},y_{n}\right)\right\}$, the data space can be divided into $m=\left\{R_{1},R_{2},\ldots R_{m}\right\}$ regions, and each region is assigned a fixed representative value $C_{i}$. The regression tree model can be represented as follows:

(1) $$f\left(X\right)=\sum_{i=1}^{m}C_{i}I(X\in R_{i}),i=1,2,\ldots m$$

Among them, $I\left(X\in R_{i}\right)$ is an indicator function, and can be represented as:

(2) $$I(X\in R_{i})=\left\{\begin{array}{c} 1,X\in R_{i}\\ 0,X\notin R_{i} \end{array}\right.$$

The specific splitting process of the regression tree can be represented as follows.

Initially, calculate the mean absolute deviation (*MAD*) for the entire dataset from the root node:

(3) $$MAD=\frac{1}{n}\sum_{i=1}^{n}\left|y_{i}-\hat{y}\right|$$

Among them, $y_{i}$ represents the actual value, $\hat{y}$ represents the predicted value, and n represents the number of the sample.

The algorithm then calculates the weighted *MAD* value after splitting for each feature and all possible split points, choosing the split point that makes the weighted *MAD* value minimum. The dataset is split into two subsets according to the chosen split point. The total *MAD* value after splitting can be expressed as:

(4) $$MAD_{T}=\frac{n_{L}}{n}MAD_{L}+\frac{n_{R}}{n}MAD_{R}$$

Among them, n is the number of samples of the current node, $n_{L}$ and $n_{R}$ are the number of samples of the left and right leaf nodes after splitting, $MAD_{L}$ and $MAD_{R}$ are the *MAD* values of the left and right leaf nodes, respectively.

Repeat the above steps for each subset, and the split is performed until each node satisfies the stopping condition for splitting. By doing so, the input space is divided into $m=\left\{R_{1},R_{2},\ldots R_{m}\right\}$ regions, resulting in the construction of the regression tree f(X).

#### 2.2.2. Pruning

Decision trees are built exclusively on training samples, enabling them to fit the training data perfectly. However, these trees can become excessively large and complex for testing samples, resulting in higher error rates. This phenomenon, known as overfitting, necessitates the simplification of the decision tree through a process called tree pruning. Common pruning techniques encompass pre-pruning and post-pruning. In practical applications, post-pruning incurs additional computational costs and complexity as it is carried out after tree construction. Hence, this study employed a combination of grid search and cross-validation to identify the optimal parameter combination. By imposing constraints on tree complexity during the construction process, overfitting can be effectively mitigated. The steps involved in this process are as follows:

1. Define Parameter Grid: The defined parameter grid includes the parameters to be searched and their possible ranges of values. These parameters are hyperparameters of the model.
2. Cross-Validation: Typically, k-fold cross-validation is employed to partition the dataset into k subsets. Each time, one subset is used as the validation set, while the remaining k − 1 subsets are used as the training set for model training and evaluation. Specifically:
  (5) $$CV_{k}=\frac{1}{k}\sum_{i=1}^{k}Score(M_{i},X_{i},y_{i})$$
  where $CV_{k}$ represents the performance evaluation metric for k-fold cross-validation, where k denotes the number of folds. $Score(M_{i},X_{i},y_{i})$ represents the performance score $X_{i}$ of the model M on the testing set in the i-th fold, corresponding to the target variable $y_{i}$.
3. Model Training and Evaluation: For each parameter combination, grid search trains the model using the specified parameter combination in each round of cross-validation and evaluates it on the validation set. The model’s performance is typically evaluated using the *MAD*.
4. Selecting the Best Parameter Combination: After completing the grid search, the parameter combination with the best performance based on the results of cross-validation is selected as the final model’s parameters.
5. Training the Final Model: Finally, the best parameter combination is used to retrain the model on the entire training dataset, resulting in the final model.

## 3. Results

**Figure 5:**
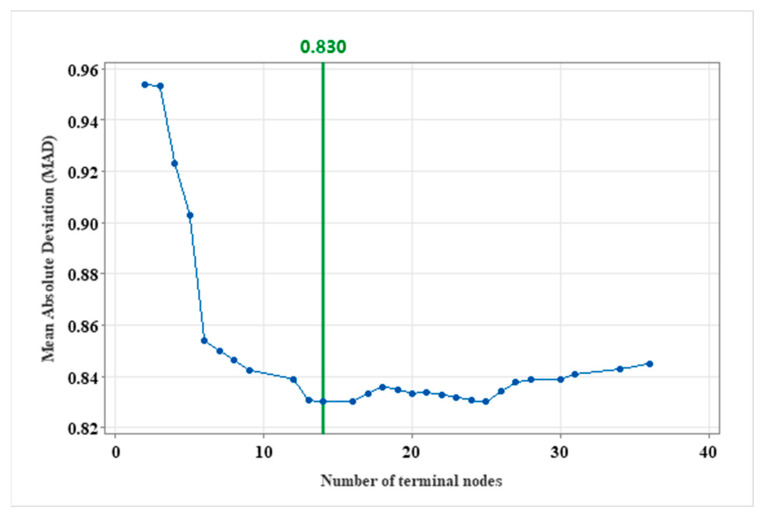
*MAD* values of different terminal nodes.

This study uses *MAD* to evaluate the modeling and predictive performance of the CART method for freeway incident duration. The specific formula for calculating *MAD* is demonstrated in Equation (3). In general, a smaller *MAD* value indicates a higher level of model accuracy. To address potential overfitting during the model construction, grid search and cross-validation are employed for the pruning process, allowing for the identification of the optimal parameter combination while constraining tree complexity. The implementation of the CART method in this study was conducted using Python programming language. To evaluate model performance under various hyperparameter combinations, a series of CART models are established with random parameter combinations. *MAD* values corresponding to different numbers of terminal nodes are shown in Figure 5. The results indicate that the model performs best with 14 terminal nodes.

### 3.1. Analysis of CART

**Figure 6:**
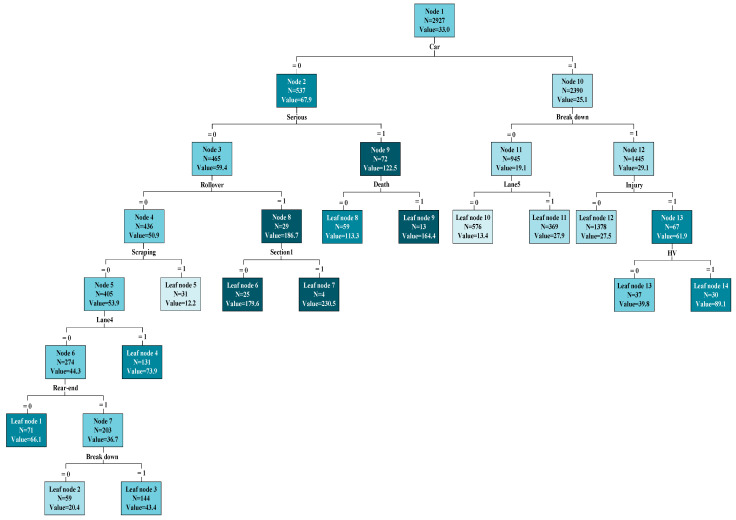
Regression tree of CART.

To classify various situations in freeway traffic incidents and devise corresponding contingency measures, this study applied the CART model with the optimal parameter combination to fit the three-year incident data of China’s Hangzhou freeway. Figure 6 shows the regression tree when the model performs optimally. The darker the color of the shadow part in the node, the longer the incident duration corresponds to the node. Examination of the regression tree revealed that the initial split of the training dataset at node 1 (the root node) was based on the “car” variable, suggesting that the presence of a car was the most significant factor influencing incident duration. Non-car incidents (i.e., car = 0, representing buses and heavy vehicles in the dataset) are guided to the left, forming node 2, while car incidents (car = 1) are directed to the right, forming node 10. Subsequent splits using the severity variable lead to milder severity incidents being guided to the left, forming node 3, and higher severity incidents being guided to the right, forming node 9.

Further splitting of node 3 occurs depending on whether incidents involve rollovers. Incidents without rollovers are directed to the left, forming node 4, while rollover incidents are steered to the right, forming node 8. Node 4 in the CART model is split based on whether incidents involve scraping, resulting in node 5 and leaf node 5. This indicates that the likelihood of scraping in non-car incidents is extremely low (approximately 0.5%), and the corresponding duration of such incidents is short (less than 15 min), making them relatively easy to deal with. Additionally, CART divides node 5 based on whether the fourth lane was closed, resulting in node 6 and leaf node 4. As seen from leaf node 4, closing the fourth lane positively affects the increase in incident duration. This is because the freeway under study mandates that heavy vehicles such as trucks should primarily use the fourth lane. Consequently, incidents in this lane are usually larger in scope and sometimes require intervention from rescue facilities such as fire trucks and trailers.

Node 6 is split based on whether incidents involve rear-end collisions, leading to leaf node 1 and node 7. Node 7 is further split into leaf nodes 2 and 3 depending on whether the vehicle involved broke down. This suggests that the duration of incidents increases significantly when the vehicle involved breaks down. The stalled vehicle cannot leave the incident site on its own, necessitating the involvement of rescue personnel, tow trucks, and other facilities, which extends the incident duration.

Likewise, CART divides node 8 based on whether the incident occurred in Section 1 (Hongken-Hongken Hub), resulting in leaf nodes 6 and 7. Notably, although incidents at the Hongken-Hongken Hub are rare, their duration has increased significantly. This is related to the traffic environment of the Hongken-Hongken Hub. Hongken connects with Xiasha Bridge (three lanes), which has a steep slope, causing truck speeds to decrease significantly. Furthermore, the road narrows from four to three lanes, reducing traffic capacity and making Hongken itself a traffic bottleneck. Consequently, rescue facilities cannot arrive in time after a vehicle rollover, prolonging the incident duration. Furthermore, incidents involving fatalities are directed to leaf nodes 8 and 9. Leaf node 9 indicates that fatalities are associated with a significant increase in incident duration due to the extra time required to summon emergency facilities such as ambulances.

Similarly, CART further splits node 10 into nodes 11–13 and leaf nodes 10–14 based on variables such as breakdown, lane five closure (close hard shoulder), injury, and heavy vehicles, as shown on the right side of the tree in Figure 4.

Examining the left side of the tree, specifically nodes 2–9 and leaf nodes 1–9, it becomes evident that incidents with higher severity, involving fatalities, or those of lower severity but involving rollovers, as well as fourth lane closures and breakdowns, exhibit significantly longer durations compared to incidents of the same type. Non-car incidents involving other types besides scraping and rear-end collisions lead to a slight increase in incident duration, warranting careful attention. Interestingly, some traffic environments or road facilities also significantly impact incident duration. Although the severity of incidents in the Hongken section is not high, road restrictions make it a bottleneck point, delaying the arrival of rescue facilities after a vehicle rollover and prolonging incident duration. On the right side of the tree, breakdowns, injuries, and the involvement of heavy vehicles all contribute to increased incident duration. Notably, the closure of the fifth lane, which is the hard shoulder, slightly increases the incident duration.

The visualization of the CART structure in Figure 4 provides an intuitive presentation of the research findings, enabling researchers to trace the paths and nodes in the tree for proposing contingency measures for different incident classifications. When incidents occur on the freeway, assessing the incident scene enables the direct implementation of relevant measures, effectively reducing incident durations. However, computers may face challenges in recognizing the graphical representation of CART, and it may be inconvenient for relevant units to consult the CART diagram after an incident. Therefore, we extracted 14 rules from the tree, presented in Table 3, to facilitate the formulation and refinement of contingency measures by relevant units and rescue personnel. These rules enable the swift identification and appropriate response to incident situations, streamlining incident management activities following their occurrence.

### 3.2. Comparison with Other Models

The prediction of incident duration via the CART method has been proven above to be of great significance in practical engineering applications. To verify its validity, XGBoost, Random Forest (RF), and AFT models were developed in the case study to predict the duration of incidents. In the XGBoost method, the optimal parameters of the model were obtained via 3-fold cross-validation; the number of trees was 102, the learning rate was 0.06, and the maximum depth was 4. For the RF model, similar to the XGBoost method, 3-fold cross-validation was used to determine the optimal setting of the parameters, resulting in 196 trees. In addition, the same variables used in the CART model were utilized to construct the AFT model, and the backward regression method in stepwise regression was employed to screen out variables with insignificant or weak significance levels. After each removal of variables, the remaining variables were re-tested, and the retained variables were all statistically significant at the 0.01 level. However, AFT models can assume different parameter distributions (such as Weibull, Log-normal, or Log-logistic) [22], and different distributions may produce different predictions. Therefore, we considered the influence of different distributions when comparing the models. The parameter estimation results of the AFT model are shown in Table 4 (taking Weibull as an example).

**Figure 7:**
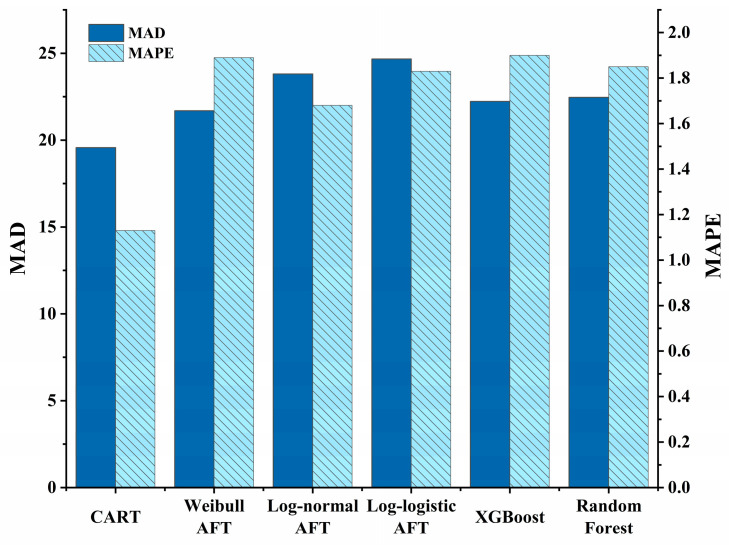
Comparison with other techniques for incident duration prediction.

To further evaluate the performance of the CART method in predicting incident duration, the prediction accuracy of the CART method was compared with that of other models. Since K-fold cross-validation was adopted in the CART method, the k − 1 subset was taken as the training set, and the remaining subset was taken as the test set. Figure 7 summarizes the results of the comparison. It is evident that, compared to other models, the developed CART method exhibited lower *MAD* and *MAPE* values. This means that the relative error between the true value and the predicted value obtained via the CART method was smaller, indicating that the accuracy of the CART method in predicting the duration of incidents was better than that of other models.

This study found that CART, as a machine learning method, was more interpretable when fitting incident duration data while maintaining accuracy. From the fitting results of the AFT model in Table 4, the influence mechanism of different variables on the duration of the incident can be found by calculating the marginal effect, which is of great significance for the analysis of incident duration. However, most of the actual incidents were caused by a combination of different factors, such as an incident between a car and a truck, which caused injury to the person involved and caused the vehicle to break down. In this way, the multifactor interaction in the incident was difficult to capture via statistical models, but it can be clearly displayed in the rules extracted from the CART method, as shown in Rule 14 in Table 3. Such interpretability is more reliable than analyzing the effect of a single variable and is more closely related to the actual incident. Similar deep interactions are analyzed and discussed in depth in Section 4.

In this case study, the CART method provided better predictions for training and test sets than the other three models. Unlike the AFT model, which must satisfy the assumption of proportional hazard, the CART approach avoided this limitation. At the same time, the CART method can effectively avoid the problem of multicollinearity. In addition, from a practical perspective, the graphical results provided by CART make it easier for analysts to understand how various factors affect incident duration. The rules extracted from it and the deep interactions captured can be more easily applied to actual traffic incidents, thus reducing the duration of incidents and the injuries caused by incidents.

## 4. Discussion

From the graphical results obtained via the CART method and the extracted rules, it is evident that real freeway incidents are caused by the interaction of various explanatory variables. The actual duration of freeway incidents is influenced by the interplay of many factors, rather than being controlled by a single variable. In the AFT and other models mentioned above, the focus is more on identifying which variables affect the duration of the incident [16,22], often ignoring potential interactions. This further demonstrates the value of the CART method in studying incident duration in practical engineering applications.

From the results in Table 3, different rules capture different levels of interaction, up to 7 levels of interaction. Rule 1 captures six levels of interaction, namely rear-end, scraping, rollover, fourth lane closure, car, and serious. However, rules 2 and 3 find a deeper interaction between the above variables and break down. The comparison found that other interactions being equal, break down increased the duration of the incident. To address this, freeway management authorities should enhance incident management systems and improve scene assessment to allocate resources more effectively and efficiently, and to clear stalled vehicles from incident sites. The interaction in rule 4 consists of five layers, namely scraping, rollover, fourth lane closure, car, and serious, and the incident duration under this rule is relatively long, with an average value of 73.9 min and a standard deviation of 95.3 min. This is because most vehicles driving in the fourth lane are heavy vehicles, and such incidents usually have a greater impact, requiring the dispatch of trailers to assist in cleaning. The incidents that occur in rule 5 are caused only by scraping and are relatively easy to deal with. As a result, the duration of the incidents is short.

Both rules 6 and 7 capture the interaction between rollover, Hongken-Hongken Hub, car, and serious, and the corresponding incident duration is extremely long. This is because the incident caused the vehicle involved to overturn, which seriously affected the surrounding traffic environment, and the intervention of the fire department is required when cleaning up. In addition, by comparing rules 6 and 7, it is found that the Hongken-Hongken Hub variable causes the incident duration to further increase. Therefore, it is necessary to widen the lanes within this section and optimize the slope of the bridge deck to address the bottleneck at Hongken-Hongken Hub, ensuring that trailers and rescue vehicles can reach the incident scene smoothly.

The interaction of car, death, and serious is found in rules 8 and 9, which show that serious incidents (which indicate the extent and severity of the incident, rather than casualties) have a positive effect on the increase in the duration of the incident, and the duration of the incident increases further if there is a fatality. These incidents usually require intervention from rescue vehicles and emergency services. Thus, it is crucial to consider the location of rescue centers and ensure that firefighting vehicles and related facilities can swiftly reach incident scenes, especially when developing contingency plans.

In rules 10 and 11, the interaction between the variables hard shoulder closure, car, and breakdown is significant, and hard shoulder closure has a positive effect on the increase in the duration of incidents. This suggests that the incident detection system for the hard shoulder lane needs improvement. To better handle longer-duration incidents resulting from such interactions, some resources previously allocated to other lanes should be diverted to the hard shoulder lane. Rule 12 shows that the interaction between the variables of car, injury, and breakdown has a significant effect on the duration of incidents. In addition, rules 13 and 14 involve interactions between multiple variables. The comparison found that incidents between cars and heavy vehicles significantly increase their duration relative to car incidents. Given these interactions, it is important to set up rescue centers to ensure that rescue vehicles can arrive at incident scenes promptly. Additionally, it is essential to coordinate the monitoring of heavy vehicles on the freeway and enhance the freeway system’s ability to accurately assess heavy vehicle incidents and initial incident sites. When developing emergency plans, considering the efficiency of medical personnel and resource allocation is crucial to minimize injuries and losses associated with these freeway incidents.

## 5. Conclusions

To facilitate the practical application of incident duration research in engineering, accurate prediction of incident duration and understanding the underlying influencing variables are crucial. This study employed the CART method, a machine learning approach, based on sensor data to predict incident duration on the China Hangzhou freeway from 2019 to 2021, with a focus on maintaining interpretability and accuracy. A tree with 14 leaf nodes was constructed, extracting 14 rules that can aid relevant organizations in formulating effective contingency measures. By activating and implementing corresponding contingency plans based on the assessment of incident scenes, the duration and impact of incidents can be significantly reduced. This research contributes to advancing the practical engineering application of incident duration research.

Comparing the CART method with XGBoost, RF, and AFT models, the results show that the CART method has higher prediction accuracy. In addition, visualization of research results and extraction rules bring convenience to analysts and are more conducive to the application of research results. More importantly, the deep interaction between different variables, up to seven, is captured in the results obtained via the CART method, which is significant because actual incidents are not influenced by a single variable. These interactions tend to prolong incidents. A key focus of this study was to analyze these interactions and propose corresponding strategies. These results further prove the value of the CART method in practical engineering applications.

## Figures and Tables

**Table 1:** Comparison of the content of related studies.

Method Type	Research	Technical Methods	References (Year)
Statistical methods	Accident delay estimation and accident duration prediction	Regression models	Garib [9] (1997)
	Freeway incident duration prediction	A time sequential methodology	Khattak [10] (1995)
	Accident duration prediction of freeway systems	Loglogistic AFT metric model	Chung [14] (2010)
	Analysis of influencing factors of incident duration	Parametric AFT models considering both fixed and random parameters	Hojati [15] (2013)
	A comparative analysis of freeway crash incident clearance time	Random parameter and latent class hazard-based duration model	Islam [16] (2021)
Machine-learning methods	Causal relationship interpreting and clearance time prediction	Bayesian Model Averaging (BMA) model	Zou [23] (2021)
	A comparative study of models for incident duration prediction	K-Nearest Neighbor (KNN) method	Valenti [26] (2010)
	Traffic incident duration prediction	Improved KNN method	Wen [27] (2012)
	Traffic incident duration prediction	Support vector regression	Wu [28] (2011)
	Incident duration prediction	A probabilistic model based on a naïve Bayesian classifier	Boyles [29] (2007)
	Sequential forecast of Incident duration	Artificial neural network models	Wei [30] (2007)
	Prediction of traffic accident duration	Artificial neural network (ANN) and support vector machine (SVM)	Yu [31] (2016)
Tree-based methods	Prediction of lane clearance time of freeway incident	M5P tree algorithm	Zhan [5] (2011)
	Incident duration prediction	Tree-based quantile regression	He [32] (2013)
	Freeway incident clearance time prediction	Gradient boosting decision trees (GBDT) model	Ma [33] (2017)

**Table 2:** Description of independent variables.

Categories	Factors	Value Set
Incident characteristics	Incident type	0 = Rear-end 1 = Collision with fixed objects 2 = Scraping 3 = Rollover 4 = Fire
	Location	0 = On road 1 = Bridge 2 = Service area 3 = Toll station 4 = Interworking
	Section	0 = Xinjie-Xiaoshan East 1 = Hongken-Hongken Hub 2 = Hongken Hub-Xinjie 3 = Xiaoshan East-Keqiao West 4 = Keqiao West-Zhangjiafan
	Vehicle involved	0 = Car 1 = Heavy vehicle 2 = Coach
	Incident severity	0 = Serious incident 1 = Others
	Incident casualty	0 = Injury 1 = Death
	Number of vehicles	0 = Single vehicle incident 1 = Two-vehicle incident 2 = Multivehicle incident
	Vehicle break down	0 = Break down 1 = Others
Temporal characteristics	Time of day	0 = Daytime 1 = AM Peak 2 = PM Peak 3 = Nighttime
		0 = Holiday 1 = Weekend
Environment characteristics	Weather	0 = Sunny 1 = Rainy 2 = Foggy and snowy
Traffic characteristics	Direction	0 = Hangzhou direction 1 = Quzhou direction 2 = Bidirectional
	Lanes closure type	0 = Hard shoulder closure 1 = Lane1 closure 2 = Lane2 closure 3 = Lane3 closure 4 = Lane4 closure
Operational characteristics	Alarm source	0 = Video surveillance 1 = Telephone report 2 = Manual patrol

**Table 3:** Classification rules extracted from the CART.

Rule Number	Rule Description	Mean	Standard Deviation
1	Rear-end = {0}, Scraping = {0}, Rollover = {0}, Lane4 = {0}, Car = {0}, Serious = {0}	66.1	87.2
2	Rear-end = {1}, Scraping = {0}, Rollover = {0}, Lane4 = {0}, Car = {0}, Serious = {0}, Break down = {0}	20.4	29.5
3	Rear-end = {1}, Scraping = {0}, Rollover = {0}, Lane4 = {0}, Car = {0}, Serious = {0}, Break down = {1}	43.4	34.7
4	Scraping = {0}, Rollover = {0}, Lane4 = {1}, Car = {0}, Serious = {0}	73.9	95.3
5	Scraping = {1}, Rollover = {0}, Car = {0}, Serious = {0}	12.2	22.4
6	Rollover = {1}, Section1 = {0}, Car = {0}, Serious = {0}	179.6	145.2
7	Rollover = {1}, Section1 = {1}, Car = {0}, Serious = {0}	230.5	99.3
8	Car = {0}, Death = {0}, Serious = {1}	113.3	77.8
9	Car = {0}, Death = {1}, Serious = {1}	164.4	54.3
10	Lane5 = {0}, Car = {1}, Break down = {0}	13.4	50.7
11	Lane5 = {1}, Car = {1}, Break down = {0}	27.9	34.6
12	Car = {1}, Injury = {0}, Break down = {1}	27.5	31.7
13	Car = {1}, HV = {0}, Injury = {1}, Break down = {1}	39.8	32.5
14	Car = {1}, HV = {1}, Injury = {1}, Break down = {1}	89.1	64.6

**Table 4:** Statistical results for the AFT model.

Variable	Coefficient	Prob. |z| > Z*	Marginal Effect
Constant	2.758 ***	<0.001	-
Objects	0.468 ***	<0.001	59.7%
Scraping	−0.976 ***	<0.001	−62.3%
Rollover	1.364 ***	<0.001	291.2%
Fire	1.292 ***	<0.001	264.0%
Hongken-Hongken Hub	−0.146 ***	<0.001	−13.6%
Hongken Hub-Xinjie	−0.304 ***	<0.001	−26.2%
Heavy vehicle	0.493 ***	<0.001	63.7%
Multivehicle	0.272 ***	<0.001	31.3%
Serious	0.859 ***	<0.001	136.1%
Break down	0.336 ***	<0.001	39.9%
Nighttime	0.120 ***	<0.001	12.7%
Rainy	0.190 ***	<0.001	20.9%
Lane2 closure	−0.161 ***	0.001	−14.9%
Lane3 closure	0.158 ***	0.009	17.1%
Lane4 closure	0.150 ***	0.001	16.2%
Sigma	0.968 ***	<0.001	-

Note: *** was statistically significant at 0.01 levels. Z* is the critical value corresponding to the significance level of 0.01.

## Data Availability

All data, models, or codes that support the findings of this study are available from the corresponding author upon reasonable request.
